# Tales of the Unexpected: The Case of Zirconium(IV) Complexes with Desferrioxamine

**DOI:** 10.3390/molecules24112098

**Published:** 2019-06-02

**Authors:** Matteo Savastano, Carla Bazzicalupi, Giovanni Ferraro, Emiliano Fratini, Paola Gratteri, Antonio Bianchi

**Affiliations:** 1 Department of Chemistry “Ugo Schiff”, University of Florence, Via della Lastruccia, 3-13, 50019 Sesto Fiorentino, Italy; matteo.savastano@unifi.it (M.S.); carla.bazzicalupi@unifi.it (C.B.); giovanni.ferraro@unifi.it (G.F.); emiliano.fratini@unifi.it (E.F.); 2 Consorzio per lo Sviluppo dei Sistemi a Grande Interfase (CSGI), University of Florence, Via della Lastruccia, 3-13, 50019 Sesto Fiorentino, Italy; 3 Department of NEUROFARBA- Pharmaceutical and Nutraceutical Section, and Laboratory of Molecular Modeling Cheminformatics & QSAR, University of Florence, Via Ugo Schiff 6, 50019 Sesto Fiorentino, Italy; paola.gratteri@unifi.it

**Keywords:** desferrioxamine, zirconium, chelates, molecular modelling, stability constants

## Abstract

The Zr^4+^ complexes with desferrioxamine (H_3_DFO) and its derivatives are the only ^89^Zr-based imaging agents for proton emission tomography (PET) that have been used so far in clinical trials. Nevertheless, a complete speciation of the Zr^4+^/H_3_DFO system in solution has never been performed and the stability constants of the relevant complexes are still unknown. Here we report, for the first time, the speciation of this system in water, performed by potentiometric titrations, and the determination of the stability constants of all complexes formed in the pH range 2.5–11.5. Surprisingly, although desferrioxamine gives rise to very stable 1:1 complexes with Zr^4+^ (log*K* = 36.14 for Zr^4+^ + DFO^3−^ = [ZrDFO]^+^), 2:2 and 2:3 ones are also formed in solution. Depending on the conditions, these binuclear complexes can be main species in solution. These results were corroborated by small-angle X-ray scattering (SAXS) and MALDI mass spectrometry analyses of complex solutions. Information on complex structures was obtained by means of density functional theory (DFT) calculations.

## 1. Introduction

In recent years, much interest has surged for the solution chemistry of Zr^4+^, since it was found that its ^89^Zr^4+^ radioisotope can be efficiently used for the medical diagnostic technique of positron emission tomography (PET). To prevent the free radionuclide from accumulating in living tissues, after delivery of the ^89^Zr-based imaging agents to a given target in vivo, it is necessary to bind the ^89^Zr^4+^ to a ligand able to form a complex that should satisfy the basic criteria of: (*i*) high thermodynamic and kinetic stability, even at very low concentrations of the radioisotope (10–100 nM), (*ii*) high biological compatibility, and (*iii*) presence of a ligand site for linkage to a targeting vector to provide specificity. Furthermore, rapid formation of the complex in mild conditions is desirable [1,2].

**Figure 1:**
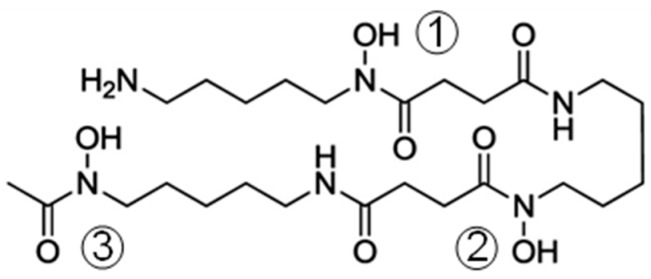
Desferrioxamine (H_3_DFO). Hydroxamate chelating units are highlighted by the numbering.

Desferrioxamine (hereafter H_3_DFO, Figure 1), the well-known Fe^3+^ chelator used for the clinical treatment of patients with iron overload [3], satisfies sufficiently well the above criteria for the preparation of ^89^Zr-based imaging agents and, accordingly, only desferrioxamine derivatives have so far been used in clinical trials [4]. Nevertheless, although the resistance of the Zr^4+^ complex with desferrioxamine toward demetallation was proven to be acceptable, the stability constant of this complex has not yet been determined. This is not the only gap, because a complete speciation of the system has never been made, i.e., we do not have a complete picture of the species that are present in solution at the various pH values, and the thermodynamic stabilities of all of them remain unknown factors. As Zr^4+^ has a greater positive charge than Fe^3+^, thus generating a stronger electrostatic attraction with the negatively charged ligand (all these are hard species, so their interactions are mainly of an electrostatic nature), the Zr^4+^ complex with the fully deprotonated DFO^3−^ molecule (containing three negatively charged, deprotonated hydroxamate groups) might be expected to be more stable than the Fe^3+^ one (log*K* in the range 30.4–30.99) [5,6,7], that is, its stability constants should be greater than 10^31^. By analogy with the Fe^3+^ complex, it is also expected that the ZrHDFO^2+^ complex is formed, in which the pendant amino group is protonated. According to density functional theory (DFT) calculations, in the latter complex, the Zr^4+^ ion is eight-coordinated, combining the six binding oxygens of HDFO^2−^ (the three hydroxamate groups) with two additional water molecules that are reported to occupy two cis positions of a distorted square antiprismatic structure [8]. Unfortunately, no crystal structures are available for these complexes, but the structure of Zr^4+^ with four bidentate hydroxamates has been a useful model for the octacoordinated environment formed by this metal ion with similar ligands [9].

Indeed, stability constants larger than 10^31^ (log*K* = 31) are a serious obstacle to the speciation of a complex system, but, in view of its importance, we liked to take up the challenge of getting around this obstacle, trying to perform a detailed speciation of the Zr^4+^–desferrioxamine system to know which species are really formed in solution and what their stabilities are.

## 2. Results and Discussion

We managed to successfully perform the speciation of the Zr^4+^/H_3_DFO system by means of potentiometric (pH-metric) titrations in aqueous solution, and we were surprised to find that there is a pH range in which the 1:1 metal-to-ligand complexes are minor components of the solution while larger aggregations (2:2 and 2:3 stoichiometries) characterize the prominent species. Complexation of Zr^4+^ by desferrioxamine suffers from some kinetic problems: potentiometric titrations showed that the complexation reaction is generally rather fast, with the exclusion of the pH range 7–9, where it becomes significantly slow (see Supplementary Materials). These kinetic problems were overcome by adopting an appropriate control on the long-term drift of electro-motive force (emf) readings during titration experiments, coupled with confirmation of results via out-of-cell batchwise experiments performed over 14 days (see Supplementary Materials). Furthermore, problems connected with the expectedly very high values of the stability constants were overcome by adopting an appropriate competing (reference) ligand. This is a standard procedure that aims to follow the transfer of the metal ion from the reference ligand to the analyzed ligand, rather than the direct coordination of the free cation (aquaion) to the analyzed ligand. This method requires that the complex species formed by the reference ligand with the specific metal ion and the relevant stability constants are known. To this purpose, we initially tested the classical competing ligand ethylenediaminetetraacetic acid (EDTA), but when we analyzed the speciation of the complexes formed with Zr^4+^ under our experimental conditions, we noticed that even EDTA forms a complicated complexation model including 1:1, 1:2, and 2:3 metal-to-ligand complexes (the species formed and their stability constants are reported in Table S1). This observation gave rise to the possibility that Zr^4+^ is capable of forming mixed-ligand complexes, that is, complexes in which one or more metal ions bind simultaneously with EDTA and desferrioxamine molecules. For this reason, the use of EDTA was abandoned and the use of other similar polydentate ligands was discarded. The suggestion of a possible alternative competing ligand came from the analysis of the hydrolytic equilibria of Zr^4+^ in water: the tetracharged cation interacts very strongly with OH^−^ anions, forming various hydroxo complexes of high stability. Then, we studied the hydrolysis of Zr^4+^ under our experimental conditions to obtain a set of data (Table S1) that was in good agreement with previous results (see Supplementary Materials) [10], which we introduced in the calculations performed by means of the HYPERQUAD [11] program to determine the stability constants of the Zr^4+^ complexes formed with desferrioxamine in the pH range 2.5–11.5 (Table 1). In an alternative model of the same validity, [Zr_2_H_2_(DFO)_2_]^4+^ can be replaced with [ZrH(DFO)]^2+^ (both are minor species that can potentially be considered to be of either mono- or di-meric nature) while the stability constants of the other complexes remain unchanged (Table S2).

**Figure 2:**
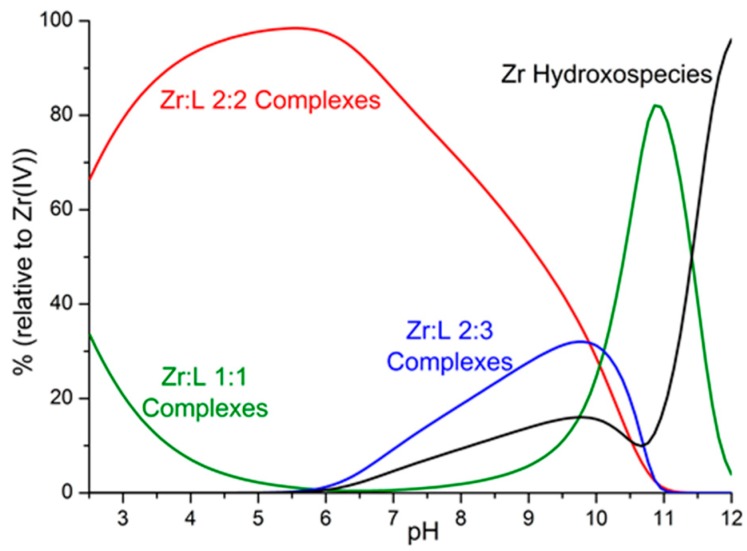
Distribution diagram of the complexes formed in the Zr^4+^–desferrioxamine system representing the overall percentages of 1:1, 2:2, and 2:3 metal-to-ligand and hydroxo complexes, obtained by means of the Hyss program [12] on the basis of the determined equilibrium constants. Desferrioxamine = L; [Zr^4+^] = [L] = 1 mM. Charges omitted.

An inspection of the data in Table 1 shows that the [ZrDFO]^+^ complex is characterized, as expected, by very high stability (log*K* = 36.14), which is largely greater than the stability of the corresponding Fe^3+^ complex (log*K* in the range 30.4–30.99). Nevertheless, as shown by Figure 2, its formation in a solution containing Zr^4+^ and H_3_DFO, both 1 mM, is minimal due to the competitive effect of the very stable 2:2 and 2:3 metal-to-ligand complexes. For simplicity, the distribution diagram in Figure 2 was drawn to show the cumulative percentages of complexes with 1:1, 2:2, and 2:3 metal-to-ligand molar ratios, respectively, along with the percentage of Zr^4+^ hydroxo species. A distribution of the individual species formed under identical conditions can be found in the Supplementary Materials (Figure S1).

As can be seen in Figure 2, 2:2 metal-to-ligand complexes are largely predominant below pH 10, while complexes with 1:1 stoichiometry become predominant only above pH 9.5, reaching a maximum of about 80% around pH 11. The strongly competitive hydroxo complexes start being formed from pH 6 to be the main species above pH 11.5, and at pH 12 they are almost the only zirconium species in solution.

**Figure 3:**
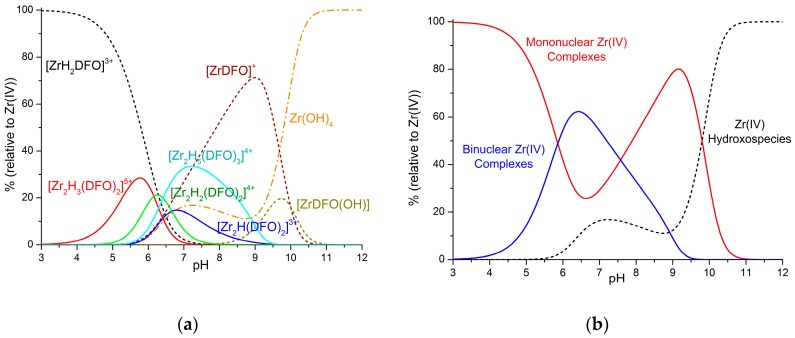
Distribution diagrams of the complexes formed in the Zr^4+^–H_3_DFO system ([Zr^4+^] = [L] = 100 nM). (**a**) Diagram of individual species. Solid lines: Binuclear species; dashed lines: Mononuclear species; dash–dot lines: Zr^4+^ hydroxo species. Only species formed with percentages of formation higher than 2% are reported. (**b**) Diagram with overall percentages of mononuclear, binuclear, and hydroxo complexes. Distribution diagrams were obtained by means of the Hyss program [14] on the basis of the determined equilibrium constants.

An obvious question at this point regards what we can expect to occur under the conditions used for the current radiolabeling protocols, which assume that 100% of the complexes are the 1:1 species with complex dilution down to the nM range and pH of about 6.5–7.5 [13,14]. A prediction of what should happen in these conditions can be made, with the help of the Hyss program [12], by using the equilibrium data reported in Table 1 and Table S2. For instance, as shown in Figure 3, the stability of the binuclear complexes is so high that they remain the prevailing complexes in solution in the 6.5–7.5 pH range, even at 100 nM concentration.

**Figure 4:**
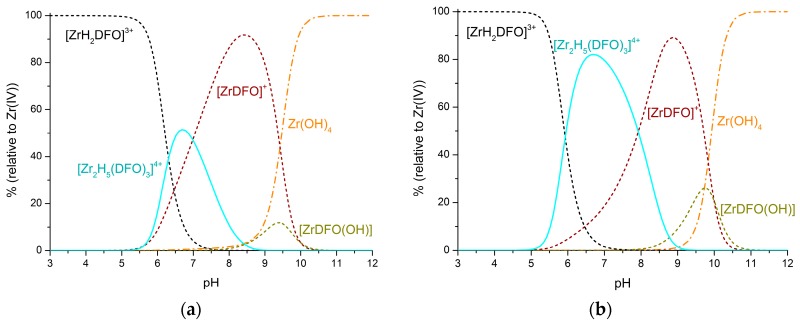
Distribution diagrams of the complexes formed in the Zr^4+^–H_3_DFO system. Solid lines: Binuclear species; dashed lines: Mononuclear species; dash–dot lines: Zr^4+^ hydroxo species. (**a**) [Zr^4+^] = 1 nm, [H_3_DFO] = 10 nM. (**b**) [Zr^4+^] = 1 nM, [H_3_DFO] = 100 nM. Distribution diagrams were obtained by means of the Hyss program [14] on the basis of the determined equilibrium constants.

By further lowering the concentration, the mononuclear species become prevalent. Nevertheless, the large excess of ligand used in radiolabeling promotes the formation of 2:3 metal-to-ligand complexes, which become increasingly abundant as the ligand-to-metal molar ratio increases. For instance, in a solution containing Zr^4+^ 1 nM and a 10-fold excess of H_3_DFO, the [Zr_2_H_5_(DFO)_3_]^4+^ complex is the main species in a narrow pH range around 6.5 (Figure 4a), but if the ligand excess is raised to 100-fold, this 2:3 species becomes largely prevalent (up to 80%) in the 6.5–7.5 pH range (Figure 4b). Conversely, in all cases (Figure 3 and Figure 4), the 1:1 species prevails in acidic media (pH < 5), where the [ZrH_2_DFO]^3+^ complex is formed, or in solutions around pH 8.5–9, where [ZrDFO]^+^ becomes predominant. Above pH 10.5, the strongly competitive hydroxo complexes are always the only zirconium species in solution.

The formation of 2:2 and 2:3 metal-to-ligand complexes was confirmed by MALDI-TOF/TOF mass spectrometry performed by using a α-cyano-4-hydroxycinnamic acid (HCCA) matrix on samples containing Zr^4+^ and H_3_DFO in 1:1 and 1:1.5 molar ratios (Figures S2–S5), which showed peaks with correct intensity distributions for [KZr_2_(DFO)_2_HCCA(−2H^+^)] (*m*/*z* 1520.87, Figures S3 and S4) and for [HKZr_2_(DFO)_3_]^+^ (*m*/*z* 1892.30, Figure S5). Only 1:1 species ([ZrDFO]^+^, *m*/*z* 647.42) were found with ESI mass spectrometry (Figure S6).

Since we did not succeed in growing crystals of the complexes suitable for X-ray analysis, information on the structures of the 1:1 and 2:2 complexes were obtained in the gas phase by means of DFT calculations at the B3LYP level of theory (MSV basis set) [15]. The terminal amine group of DFO was assumed to be protonated in [Zr_2_H_2_(DFO)_2_]^4+^, according to the equilibrium data, while both unprotonated and protonated forms were considered for the 1:1 complex.

**Figure 5:**
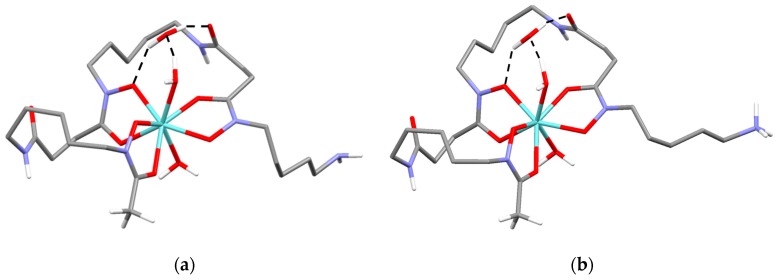
Calculated lowest energy conformers of: [ZrDFO]^+^ (**a**), [ZrHDFO]^2+^ (**b**).

The lowest energy conformer of the 1:1 [ZrDFO]^+^ complex shows the metal ion in an eight-coordinated environment formed by three chelating units of DFO^3−^ and two water molecules (Figure 5a). The two water molecules lie in trans positions, in contrast to the cis arrangement found in the previously cited study [8]. An additional non-coordinated water molecule stabilizes the complex, forming an H-bond network mainly involving one chelating ring. Protonation of the terminal amine group would not produce substantial modification of this structure (Figure 5b).

**Figure 6:**
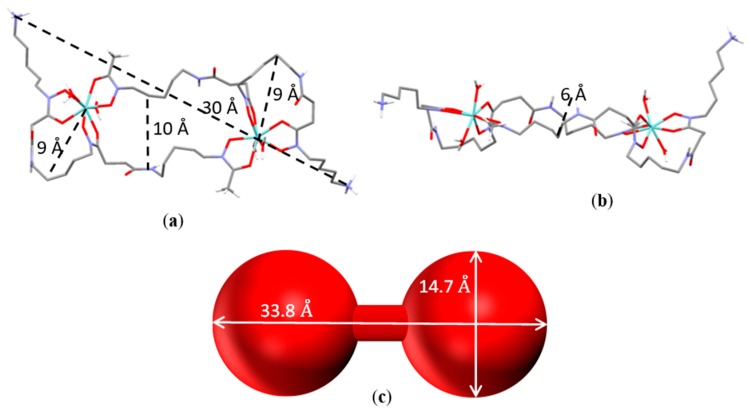
Calculated lowest energy conformer of [Zr_2_H_2_(DFO)_2_]^4+^: Top (**a**) and lateral (**b**) views. (**c**) The barbell model of the main complex obtained from small-angle X-ray scattering (SAXS) measurements in solution ([Zr^4+^] = [H_3_DFO] = 0.08–0.008 M).

In regards to the 2:2 complex, the shape of the calculated lowest energy conformer can be approximately described as a barbell, with the zirconium centers and their coordination environments defining the two globular end-caps (Figure 6a,b).

Each Zr^4+^ ion is coordinated by the chelating units 1 and 2 of a desferrioxamine molecule (Figure 1), and the overall eight-coordination environment is completed by the chelating unit 3 of the second ligand and by two water molecules in trans positions (Zr–O distances in the range 2.1–2.3 Å). The diameter of the end-caps, measured as twice the distance from the metal center of the furthest atom in the chelating ring given by the units 1 and 2, is about 18 Å, while the bar of the barbell is about 10 Å large (Figure 6a) and 6 Å thick (Figure 6b). The pendant arms protrude outside and contribute to define the overall length of this binuclear complex, which is about 30 Å.

Information on the structure of the 2:2 complex in solution was obtained by means of small-angle X-ray scattering (SAXS) analysis of aqueous solutions of Zr^4+^ and DFO in a 1:1 molar ratio (0.08 M and 0.008 M) at pH 4, that, according to the equilibrium data in Table 1 (or Table S2), should contain 2:2 species in about 90%. Simulated scattering profiles were obtained for 1:1 and 2:2 metal-to-ligand complexes, on the basis of the calculated structures reported above, by means of the CRYSOL [16] program. Comparison of the simulated profiles with the experimental curves clearly shows that the best agreement is given by the curves of the 2:2 complex. Furthermore, we found that the experimental scattering patterns can be appropriately modelled by using a barbell form factor [17]. The two globular end-caps of the barbell correspond to the zirconium atoms, which are responsible for the main contribution to the measured scattering (Figure 6c). Fitting of the SAXS data furnished the length (4.4 ± 0.3 Å) and the diameter (2.5 ± 1.6 Å) of the bar, the diameter (14.7 ± 0.2 Å) of the globular end-caps of the barbell, and the overall barbell length (33.8 ± 0.8 Å), which are in good agreement with the lowest energy conformer obtained for [Zr_2_H_2_(DFO)_2_]^2+^ by means of DFT calculations.

## 3. Conclusions

In conclusion, we managed to perform, for the first time, the speciation of the Zr^4+^–desferrioxamine system and to determine the stability constants of the complexes formed in water in the 2.5–11.5 pH range. Surprisingly, in contrast to the general belief that desferrioxamine forms only 1:1 complexes with Zr^4+^, potentiometric and SAXS measurements in solution, corroborated by MALDI mass spectrometry, showed that the main complexes formed in a large pH region are characterized by larger aggregation, with 2:2 and 2:3 metal-to-ligand stoichiometry. The stabilities of these binuclear complexes are so high that they remain the principal species even in very diluted solutions, under pH and reagent concentrations approaching the conditions used for radiolabeling. Of course, this does not necessarily mean that this tendency to dimerization is maintained even after the terminal amine groups of desferrioxamine molecules have been functionalized, e.g., when they have been linked to monoclonal antibodies for the preparation of positron ^89^Zr-based emitters for PET imaging. However, in light of this new information, this point might deserve specific consideration. It is noteworthy that, as early as 1992, W.E. Meijs et al., after analyzing the UV spectra of Zr^4+^–DFO mixtures, commented “when the U.V. spectra of the reaction mixtures with an excess of desferal were corrected for this desferal excess, sometimes an overcorrection was observed”, and suggested as an explanation of this phenomenon the interaction of Zr^4+^ with hydroxamate groups of two different DFO molecules [18], which is in agreement with the formation of the 2:3 metal-to-ligand complexes found by us.

The equilibrium data reported here allow one to envisage the complexation model before labelling under their own labelling conditions, and to choose the more appropriate conditions (pH, concentrations, ligand excess) to have the desired reactive species in solution. Furthermore, a certain slowness, requiring up to 2 h for equilibration, was found for complex formation occurring in the pH region useful for current radiolabeling protocols. We believe that this new body of information will be of interest for people working with ^89^Zr-based radiopharmaceuticals, certainly from a theoretical perspective and, perhaps, also from applicative points of view, and will stimulate new concepts in the design of chelating agents for Zr^4+^ coordination.

## Figures and Tables

**Table 1:** Equilibrium constants of the complexes formed by Zr^4+^ with desferrioxamine (H_3_DFO) in 0.1 M NMe_4_Cl at 298.1 K.

Equilibrium	log*β*
Zr^4+^ + DFO^3−^ = [ZrDFO]^+^	36.14(9) ^1^
Zr^4+^ + DFO^3−^ + 2H^+^ = [ZrH_2_DFO]^3+^	49.06(6)
Zr^4+^ + DFO^3−^ + H_2_O = [Zr(DFO)OH] + H^+^	26.15(4)
2Zr^4+^ + 2DFO^3−^ + H^+^ = [Zr_2_H(DFO)_2_]^3+^	86.3(1)
2Zr^4+^ + 2DFO^3−^ + 2H^+^ = [Zr_2_H_2_(DFO)_2_]^4+^	92.9(2)
2Zr^4+^ + 2DFO^3−^ + 3H^+^ = [Zr_2_H_3_(DFO)_2_]^5+^	99.1(1)
2Zr^4+^ + 3DFO^3−^ + 5H^+^ = [Zr_2_H_5_(DFO)_3_]^4+^	134.1(1)
2Zr^4+^ + 3DFO^3−^ + 6H^+^ = [Zr_2_H_6_(DFO)_3_]^5+^	138.0(1)

^1^ Values in parentheses are standard deviations of the last significant figures.

## Supplementary Materials

The following are available online. Experimental details of potentiometric measurements, computational studies, MALDI and ESI analyses, small-angle X-ray scattering (SAXS) experiments, Table S1: equilibrium constants of the complexes formed by Zr^4+^ with EDTA, Table S2: equilibrium constants of the complexes formed by Zr^4+^ with DFO, Figure S1: distribution diagrams for the Zr^4+^-H_3_DFO system ([Zr^4+^] = [L] = 1mM) in aqueous solution.
