# Supplementary material for: Tales of the Unexpected: The Case of Zirconium(IV) Complexes with Desferrioxamine
Source: Molecules. 2019 Jun 2;24(11):2098. doi: 10.3390/molecules24112098 (PMC6600682; doi:10.3390/molecules24112098)
Supplement: Supplementary file 1 [file molecules-24-02098-s001.pdf]

Supplementary Materials

Tales of the unexpected: the case of zirconium(IV) complexes with desferrioxamine

Matteo Savastano,[a] Carla Bazzicalupi,[a] Giovanni Ferraro,[a,b] Emiliano Fratini,[a,b] Paola Gratteri,[c] Antonio Bianchi,[a]

[a] Department of Chemistry “Ugo Schiff”, University of Florence, Via della Lastruccia 3, 50019, Sesto Fiorentino, Italy.

[b] Consorzio per lo Sviluppo dei Sistemi a Grande Interfase (CSGI), Via della Lastruccia 3, 50019 Sesto Fiorentino, Italy.

[c] Department of Pharmaceutical Sciences, and Laboratory of Molecular Modeling Cheminformatics & QSAR, University of Florence, Via Ugo Schiff 6, 50019 Sesto Fiorentino, Italy.

Table of Contents

Potentiometric measurements ……………………………………………………………………………………… S3

Table S1. Equilibrium constants of the complexes formed by Zr^{4+} with EDTA ………………….. S6

Table S2. Equilibrium constants of the complexes formed by Zr^{4+} with DFO …………………… S7

Figure S1. Distribution diagrams for the Zr^{4+}-H_3DFO system in aqueous solution……………. S8

Computational studies …………………………………………………………………………………………….. S9

MALDI analyses ……………………………………………………………………………………………………….. S13

ESI analysis …… S17

Small-angle X-ray scattering (SAXS) experiments …………………………………………………………. S18

References ……. S24

Potentiometric measurements

Preparation of Zr(IV) stock solution

Commercial $ZrCl_4$ (5 g, 99.99%, Sigma-Aldrich) was dissolved in 200 ml 0.1 M HCl aqueous solution: such manipulation has been done carefully into a glove box.

The obtained stock solution (0.1 M approx.) has been standardized against 1.000 M EDTA solution (commercial Titriplex kit of Na_2-EDTA from Merck) using standard procedures (50° C, xylenol orange indicator).

The result of 7 independent titrations experiments provided a 0.1079(1) mol/kg concentration for the stock solution.

Standard procedure adopted for Zr(IV)-Desferrioxamine and Zr(IV)-hydroxo potentiometric titrations

Stock solution of $ZrCl_4$ in 0.1 M HCl (cf. dedicated section) and solid desferrioxamine salt (mesylate, high purity as intended for medical usage, from Novartis), have been used in the measurements.

Potentiometric (pH-metric) titrations, used to determine ligand protonation and complex stability constants, were performed in 0.1 M NMe_4Cl at 298.1 ± 0.1 K using an automated apparatus and a procedure previously described.[1] The combined Metrohm 6.0262.100 electrode was calibrated as a hydrogen-ion concentration probe by titrating previously standardized amounts of HCl with CO_2-free NaOH solutions and determining the equivalent point by Gran's method,[2] which gives the standard potential, E°, and the ionic product of water (pKw = 13.83(1) in 0.1 M NMe_4Cl at 298.1 K). Calibration was performed before and after each measurement to ensure constancy of cell parameters. Computer program HYPERQUAD[3] was used to calculate ligand protonation and complex stability constants from potentiometric data.

Zr(IV) and desferrioxamine concentrations ranged between $5 \cdot 10^{-4}$ M and 2 mM. Various metal to ligand ratios from 1:1 up to 1:2 have been explored. Measurements can be safely conducted up to the 10.0-11.5 pH range without precipitation of solids (significant excess of ligands is effective in preventing the formation of insoluble Zr(IV) hydroxospecies, allowing to safely extend the titration range in alkaline media).

Despite relatively high chloride concentration, expected to limit the formation and especially the polymerization of Zr(IV) hydroxospecies (compared to weakly coordinating nitrate or

perchlorate media), all solutions intended for measurements were left to equilibrate for 1 h inside the potentiometric cell. This is largely unnecessary (actual potential variations tied to the initial complexation of Zr(IV) to desferrioxamine last no more than 30 minutes), yet it is done to allow the strong complexation of the metal with the ligand to remove any traces of soluble polynuclear Zr(IV) species.

Acidic branch of the titrations is rather fast: it can be reliably recorded by waiting 3-5 minutes per point between titrant injection and emf readings (0.05 mV maximum allowed standard deviation, 0.1 mV/min maximum allowed drift). Anyhow, this has been also checked with a holding time of 20 minutes per point, showing no appreciable differences with faster experiments.

As we approach pH 7, starting from about pH 5 and ending above pH 9, the system starts to become gradually slower from a kinetic standpoint.

In this region measurement times were manually adjusted to ensure the collection of equilibrium emf values.

In the slowest region, pH 7.0-9.0, measurements have been conducted with long holding times (60 to 90 minutes) between injection and emf readings, for which quality parameters have been made far more stringent. For each point an emf value was measured every 18s, a 0.05 mV maximum standard deviation and a 0.01 maximum drift was allowed on each set of 10 readings (this would correspond to a maximum drift of 0.03 mV/min, a 3 times lower threshold with respect to the above to ensure reaching equilibrium). If a set of 10 readings does not satisfy the specified parameters, the system is allowed to acquire further readings up to a maximum of 100. Overall, this means that for a 90 minutes holding time point, a titration point can be collected in 93 minutes (90 minutes holding time + (10 readings*18 seconds/reading)/60 = 93 minutes), if the system is at the equilibrium when the emf readings begin, or in up to 120 minutes (90 minutes holding time + (100 readings*18 seconds/reading)/60 = 120 minutes), if equilibrium is reached only at the 100th reading.

After pH 9.5, measurements become relatively fast again, and points can be collected within a 10-20 minutes timeframe each.

Both complete and partial curves, especially in the slow region, have been recorded through this methodology.

Measurements for the determination Zr(IV) hydroxo complexes were performed with the same apparatus. Five titrations were performed in the pH range 2-3.7 adopting equilibration times from 5 to 20 minutes between titrant (0.1 M NMe_4Cl) additions. Each curve was separately treated with

the computer program HYPERQUAD to obtain coincident sets of hydrolysis constants. Then, the five curves were merged together and analysed to give the constants for hydrozo Zr(IV) complexes reported in Table S2. For the sake of comparison, in the same table, we have reported literature values previously obtained for the same equilibria. As can be seen, there is a difference in the first hydrolysis constants that can be ascribed to the competition of OH^- anions with coordinated Cl^- anions, that are in high concentration in our medium (0.1 M NMe_4Cl). This competition vanishes in the last substitution equilibria as the interaction of Zr(IV) with Cl^- anions become weaker and weaker (Table S2).

Equilibrium constants for Cl^- coordination to Zr^{4+} (Table S2) were introduced in all calculations involving potentiometric data for Zr^{4+} complexation. For all systems, measurements for which significant drift of instrumental cell parameters before and after recording titration curves has been observed were discarded.

Procedure adopted for Zr(IV)-Desferrioxamine batchwise potentiometric titrations

To consolidate the potentiometric information, ensuring both to have reached equilibrium and avoided instrumental issues over the course of long measurements, 15 different Zr(IV)-Desferrioxamine solutions were prepared for batchwise measurement, spanning the 3.1-10.2 pH range and selectively exploring the kinetic slow region from pH 7.0-9.5. 11 Solutions were prepared in a 1:1 metal to ligand ratio ratio and 4 in a 1:2 metal to ligand ratio instead.

All sample handling and storing has been done as carefully as possible: samples have been sealed under nitrogen, to avoid sample contamination form CO_2, and stored at 298(1) K.

Measurements were made after a holding time of two weeks. No significant differences between batchwise and standard potentiometric titrations were detected. Batchwise data points can be used in the HYPERQUAD refinement of the final stability constants together with standard titrations data, furnishing the same results (identical within the experimental errors).

Table S1. Equilibrium constants of the complexes formed by Zr^{4+} with ethylenediaminetetraacetic acid (H_4EDTA) in 0.1 M NMe_4Cl at 298.1 K. Constants were obtained by adopting the same procedure described for the Zr-DFO system.

Equilibrium	log β
$Zr^{4+} + EDTA^{4-} = [ZrEDTA]$	31.1(1)
$Zr^{4+} + EDTA^{4-} + H^+ = [ZrHEDTA]^+$	33.4(1)
$Zr^{4+} + EDTA^{4-} + H_2O = [Zr(EDTA)OH]^- + H^+$	24.22(1)
$Zr^{4+} + 2EDTA^{4-} + H^+ = [ZrH(EDTA)_2]^{3-}$	46.4(1)
$Zr^{4+} + 2EDTA^{4-} + 2H^+ = [ZrH_2(EDTA)_2]^{2-}$	52.7(1)
$Zr^{4+} + 2EDTA^{4-} + 3H^+ = [ZrH_3(EDTA)_2]^-$	57.5(1)
$2Zr^{4+} + 3EDTA^{4-} + 4H^+ = [Zr_2H_4(EDTA)_3]$	98.1(2)

Table S2. Synopsis of the two different sets of stability constants providing an equally good fitting of the experimental data. All stability constants are equal within the experimental error (figure in parentheses) except the ZrHDFO (111) and $Zr_2H_2DFO_2$ (222) species, which can potentially be considered either of mono- or dimeric nature. Protonation constants of DFO^{3-} and hydrolysis constants of Zr^{4+} are also provided. All measurements were made in 0.1 M NMe_4Cl at 298.1 K. [a]) Taken from Baes, C.F.; Mesmer, R.E. The hydrolysis of cations. John Wiley, New York, **1976**. [b]) Taken from Solovkin, A.S. *Zhur. Neorg. Khim.* **1957**, *2*, 611.

Equilibrium	Log β 222 Model	Log β 111 Model
$Zr^{4+} + DFO^{3-} = [ZrDFO]^{+}$	36.02(9)	36.02(9)
$Zr^{4+} + DFO^{3-} + H^{+} = [ZrHDFO]^{2+}$	_	44.7(1)
$Zr^{4+} + DFO^{3-} + 2H^{+} = [ZrH_2DFO]^{3+}$	49.06(6)	49.05(6)
$Zr^{4+} + DFO^{3-} + H_2O = [Zr(DFO)OH] + H^{+}$	26.15(4)	26.14(4)
$2Zr^{4+} + 2DFO^{3-} + H^{+} = [Zr_2H(DFO)_2]^{3+}$	86.3(1)	86.2(1)
$2Zr^{4+} + 2DFO^{3-} + 2H^{+} = [Zr_2H_2(DFO)_2]^{4+}$	92.9(2)	_
$2Zr^{4+} + 2DFO^{3-} + 3H^{+} = [Zr_2H_3(DFO)_2]^{5+}$	99.1(1)	99.0(1)
$2Zr^{4+} + 3DFO^{3-} + 5H^{+} = [Zr_2H_5(DFO)_3]^{4+}$	134.1(1)	134.0(1)
$2Zr^{4+} + 3DFO^{3-} + 6H^{+} = [Zr_2H_6(DFO)_3]^{5+}$	138.0(1)	138.0(1)
Additional equilibria for both models	log*K*	
$DFO^{3-} + H^{+} = HDFO^{2-}$	10.70(2)	
$HDFO^{2-} + H^{+} = H_2DFO^{-}$	9.72(2)	
$H_2DFO^{-} + H^{+} = H_3DFO$	8.93(3)	
$H_3DFO + H^{+} = H_4DFO^{+}$	8.35(3)	
$Zr^{4+} + H_2O = ZrOH^{3+} + H^{+}$	-1.71(6)	-0.36[a]
$Zr^{4+} + 2H_2O = Zr(OH)_2^{2+} + 2H^{+}$	-3.81(3)	-2.81[a]
$Zr^{4+} + 3H_2O = Zr(OH)_3^{+} + 3H^{+}$	-6.67(4)	-6-43[a]
$Zr^{4+} + 4H_2O = Zr(OH)_4 + 4H^{+}$	-11.51(9)	-11.04[a]
$Zr^{4+} + Cl^{-} = ZrCl^{3+}$	0.92[b]	
$ZrCl^{3+} + Cl^{-} = ZrCl_2^{2+}$	0.40[b]	
$ZrCl_2^{2+} + Cl^{-} = ZrCl_3^{+}$	0.19[b]	
$ZrCl_3^{3+} + Cl^{-} = ZrCl_4$	-0.33[b]	

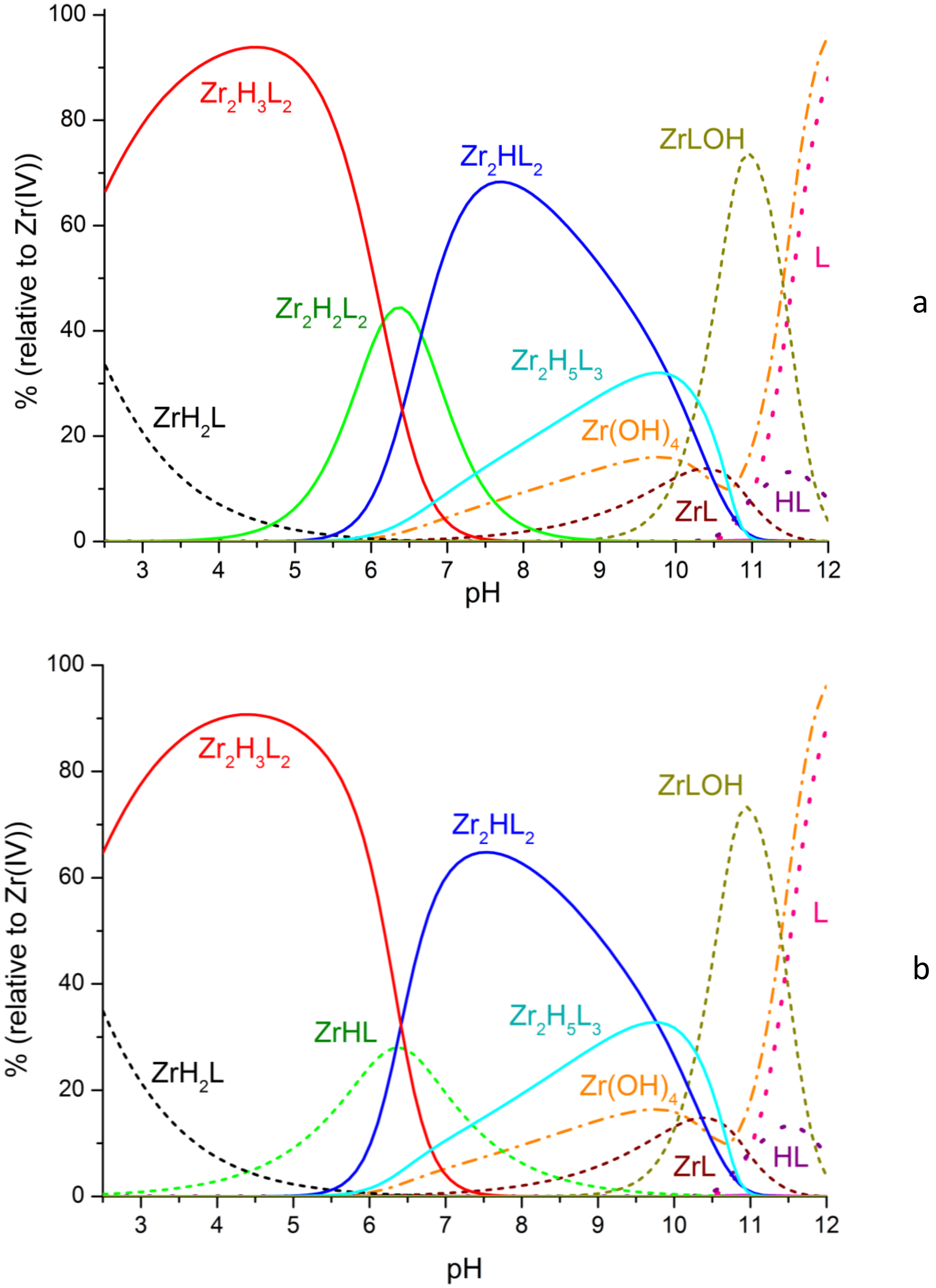

Figure S1. Distribution diagrams for the Zr^{4+}-desferrioxamine system (DFO^{3-} = L , charges omitted for simplicity) in aqueous solution. Solid lines: polynuclear species; dashed lines: mononuclear species; dotted lines: free ligand species; dash-dot lines: Zr^{4+} hydroxospecies. [Zr^{4+}] = [L] = 1mM. a) Diagram including the 2:2:2 $Zr_2H_2L_2$ species. b) Diagram including the alternative 1:1:1 ZrHL species.

Computational studies

Experimental details

1:1 and 2:2 Zr^{4+}:DFO complexes were analysed. For each system, the nitrogen atom of the pendant arm was considered both protonated (ammonium) or deprotonated (amine).

Starting coordinates for $[ZrDFO]^{+}$ were obtained by properly modifying the coordinates of the DFO/Fe^{3+} complex, reported in the literature,[4] to replace the iron centre with Zr^{4+} and to expand to 8 the metal coordination number. Several hypotheses were considered saturating the coordination sphere with the nitrogen atom of the terminal amine group and one water molecule (*cis*N,O monomer), or with two *cis* (*cis*O,O monomer) or two *trans* (*trans*O,O monomer) water molecules.

Starting coordinates for $[Zr_2H_2(DFO)_2]^{4+}$ were first manually built from the crystal structure of the iron complex.[4] Two mononuclear complexes were joined together by symmetrically moving, one at the time, the chelating unit 1 or 3 (Chart S1) from one metal centre to the other one, so obtaining two different bridged conformations. These strained conformations were then relaxed

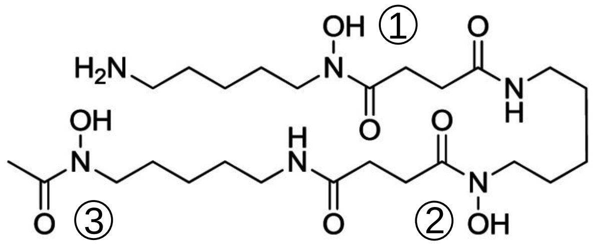

Chart S1. DFO (Desferrioxamine)

by short molecular dynamic procedures (Macromodel,[5] OPLS, T=400 K, step=1.5 fs, equilibrating time = 1 ps, running time = 10 ps; all coordination bonds constrained to the starting values; Zirconium(IV) ions replaced by Zn^{2+} ions to make the OPLS forcefield work). As for the mononuclear complex, the coordination sphere of each zirconium ion was expanded to 8 by coordination of two water molecules, while the nitrogen atom of the terminal amine group was maintained protonated.

The possible effect of an additional not-coordinated water molecules was taken into account.

Each 1:1 or 2:2 complex was fully minimized with Jaguar[5] at the DFT/B3LYP level of theory, MSV basis set. The nature of stationary points as true minima was checked by frequency calculations.

Analysis of results and structural considerations

As no preliminary experimental structural information was available for DFO/Zr^{4+} complexes, we analysed the crystal structures of Zr(Me-AHA)$_4$ (Me-AHA = N-methyl acetohydroxamate)[6] where each AHA group behaves as a bidentate chelating unit and the metal centre is 8-coordinated. Three of the four chelating units approximately describe a propeller around the metal centre, while the fourth one approaches the zirconium ion along the propeller axis. Notably, the propeller disposition of the AHA groups in Zr(Me-AHA)$_4$ resembles that assumed by the chelating units of DFO complexes with Fe^{3+} in all reported crystal structures.[4,7] In fact, in these complexes DFO adopts very similar conformations, each having the three chelating units disposed as a propeller around the hexa-coordinated Fe^{3+} ion, with the protonated ammonium arm protruding outside. Consistently, the iron complex having the lowest agreement factor R (ref. 1) was chosen as starting structure to build the $[ZrDFO]^+$ complex. Several hypotheses were considered to expand to 8 the overall coordination number, saturating the coordination sphere by the amine arm and one water molecule (*cis*N,O), two *cis* (*cis*O,O) or two *trans* (*trans*O,O) water molecules. Moreover, since the Zr(Me-AHA)$_4$ crystal structure is highly hydrated, the possible effect of an additional not-coordinated water molecule was taken into account.

Interestingly, the (*trans*O,O) adduct converged, being the lowest energy species (Chart S2a), and maintained the overall 8-coordination (Zr-O distances in the range 2.1-2.3 Å), unlike the results of a previous study where one of the two *trans* water molecules detaches from the metal ion leaving a 7-coordinated complex.[8] The not coordinated water molecule was found to stabilize the complexes forming H-bond networks mainly involving one chelate ring. Protonation of the terminal amine group did not produce substantial modification of the complex structure (Chart S2b).

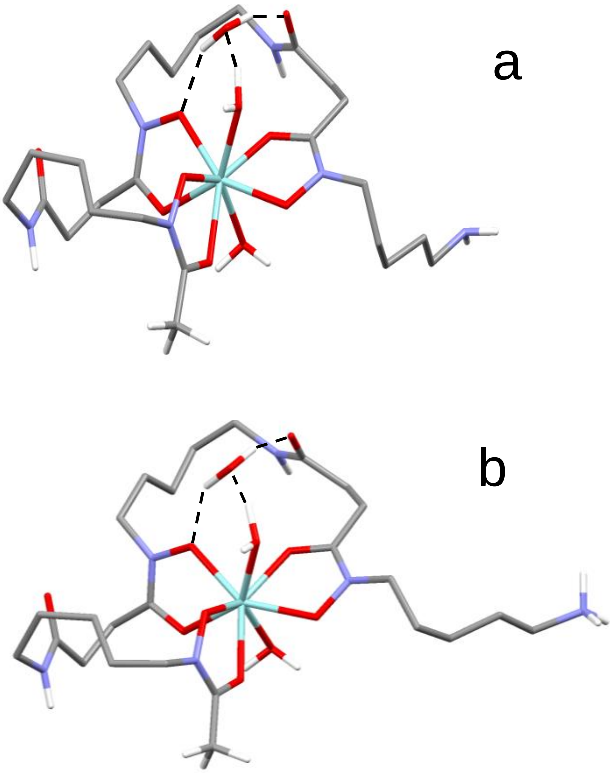

Chart S2. Calculated lowest energy conformers of $[ZrDFO]^{+}$ (a) and $[ZrHDFO]^{2+}$ (b) complexes.

As the 2:2 complex is concerned, the lowest energy conformer of $[Zr_2H_2(DFO)_2]^{4+}$ is shown in Chart S3. The complex can be approximately described as a barbell, with the zirconium centres and their coordination environments defining the two globular end-caps of the barbell. Each metal ion is coordinated by the chelating units 1 and 2 of a DFO molecule (Chart S1), the 8-coordination environment being saturated by the chelating unit 3 of the second ligand and by two coordinating water molecules in *trans* position (Zr-O distances in the range 2.1-2.3 Å). Considering the chelating ring given by the units 1 and 2, the farthest atom lies about 9 Å from the metal centre and this value can be approximately considered the radius of each globular end-cap of the barbell. Each complex is additionally stabilized by the H-bond network given by one not coordinated water molecule, analogously to the 1:1 $[ZrDFO]^{+}$ complex. The bar of the barbell, about 10 Å large and 6 Å thick, is instead constituted by the two symmetric bridges connecting the two metal centres, which lie about 14 Å apart from each other.

The protonated pendant arms protrude outside and contribute to define the overall length of this binuclear complex, which is about 30 Å.

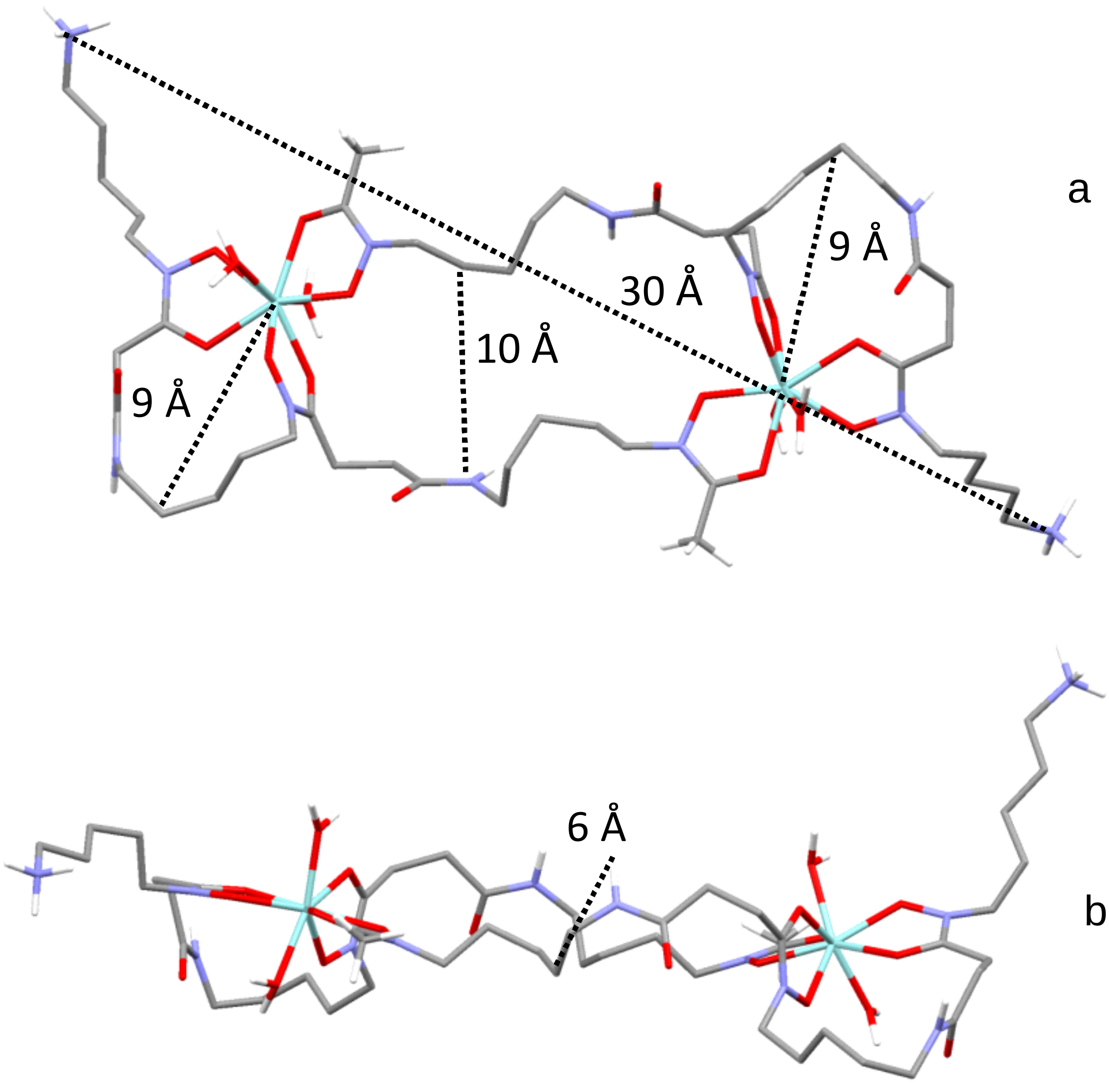

Chart S2. Calculated lowest energy conformer of $[Zr_2H_2(DFO)_2]^{4+}$: (a) top view, (b) lateral view.

MALDI analyses

A MALDI-TOF/TOF spectrometer Ultraflex III (Bruker Daltonics, Bremen, Germany) set up in Reflectron positive mode was used to perform the analyses. The spectra were acquired in the mass range of 100-2000 m/z and processed using Flex Analysis software 3.0 from Bruker Daltonics. For the analyses of the sample 1 µl of a 1:1 solution of the matrix (10 g/L α-cyano-4-hydroxycinnamic acid (HCCA) in 70/30 acetonitrile/0.1% TFA) and of the sample was spotted on a target plate and allowed to dry. The spectra obtained with 1:1 (pH 4) and 1:1.5 (pH 9) Zr:DFO samples are reported in Figures S2-S5. Assignment of some of the signals to polynuclear Zr species is rendered unambiguous due to Zr isotopic fingerprint, i.e. observed peak patterns could not be otherwise reproduced, especially with light atoms (C, N, O, H), without establishing the simultaneous presence of more than one metal centre in such species.

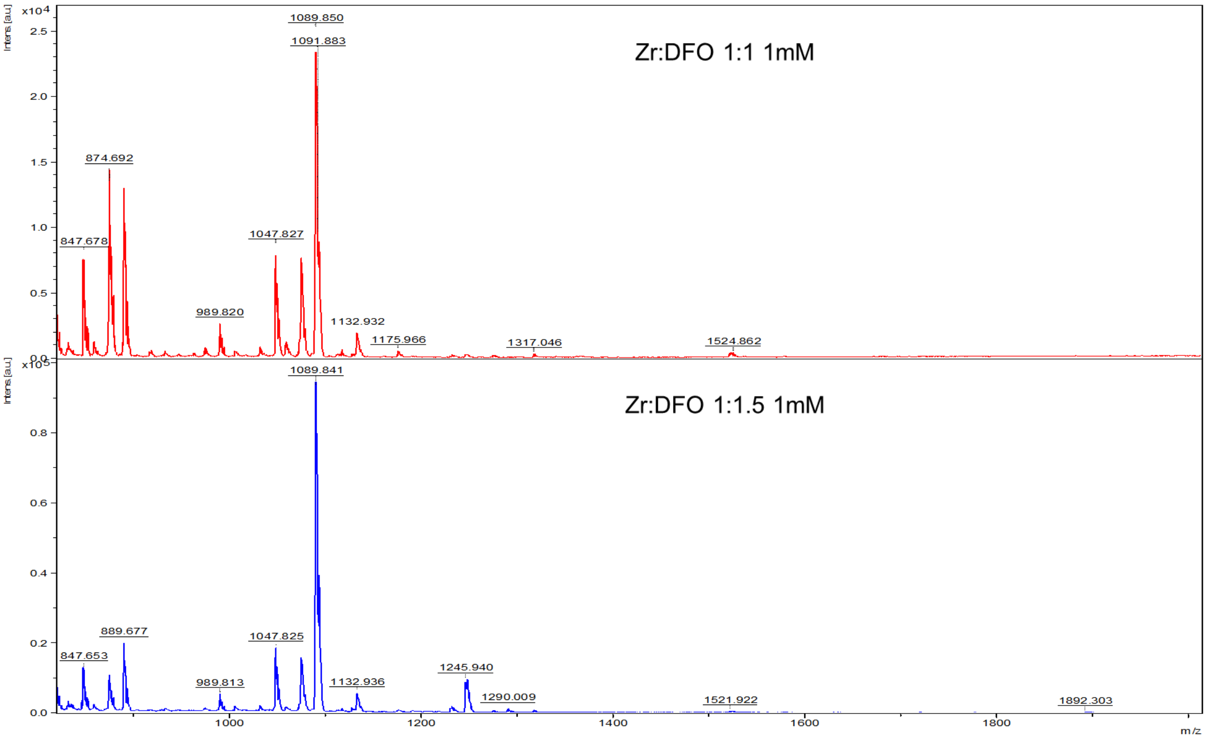

Figure S2. MALDI-TOF/TOF full spectra of the Zr:Desferrioxamine system in different conditions 1:1 and 1:1.5 meant to highlight the formation of 2:2 and 2:3 species. Main peak: $[NaZr(DFO)(HCCA)(HCCA)(-H^{+}).CH3CN]^{+}$, $C_{47}H_{61}N_9O_{14}NaZr$, calculated m/z 1088.83.

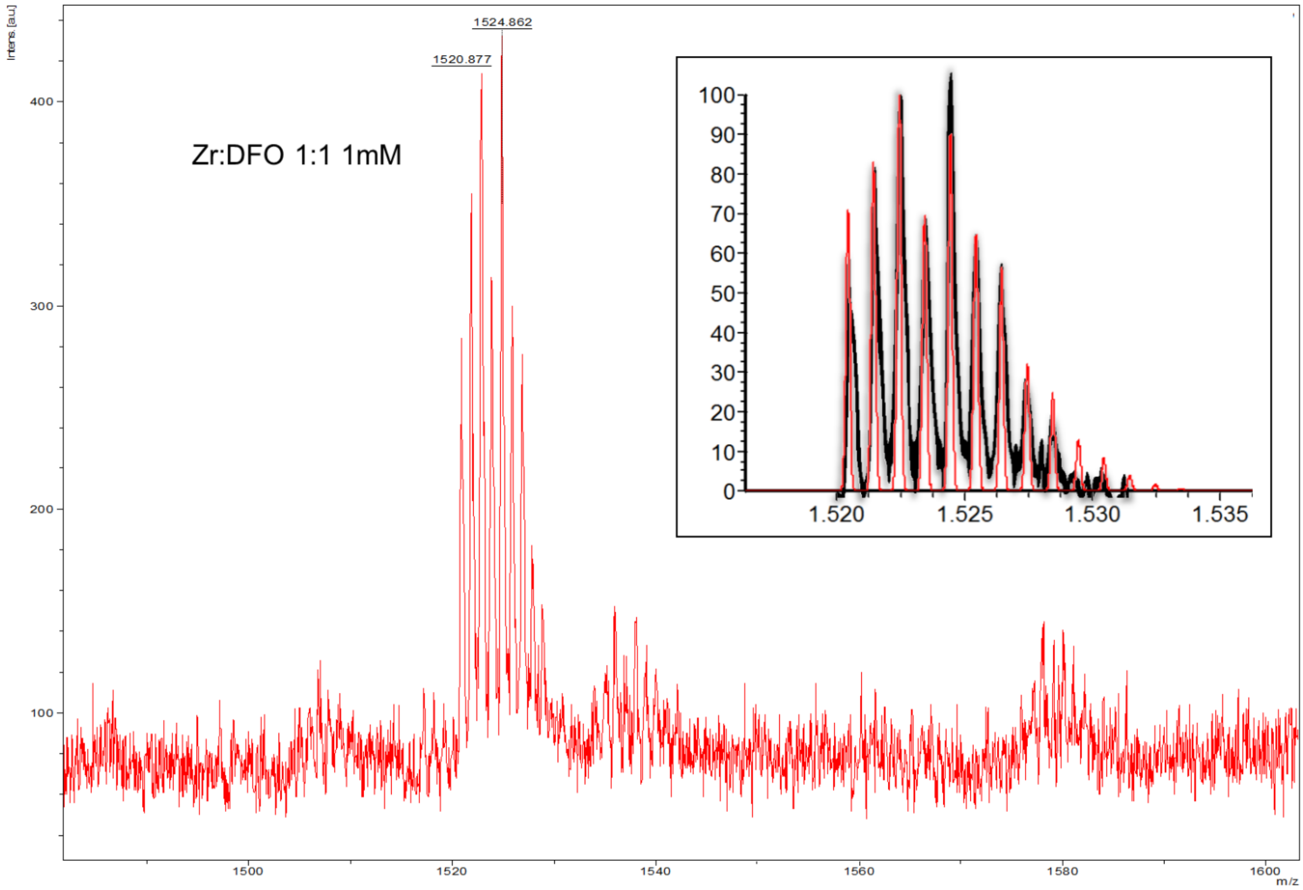

Figure S3. Detail of MALDI-TOF/TOF spectra of Zr:Desferrioxamine 1:1 highlighting the peak at m/z 1520.87. Inset: experimental (black) vs calculated (red) intensity distribution for $[KZr_2(DFO)_2HCCA(-2\ H^+)]^+$, $C_{60}H_{95}N_{13}O_{19}KZr_2$, calculated m/z 1520.46. HCCA (α-cyano-4-hydroxycinnamic acid) from the matrix.

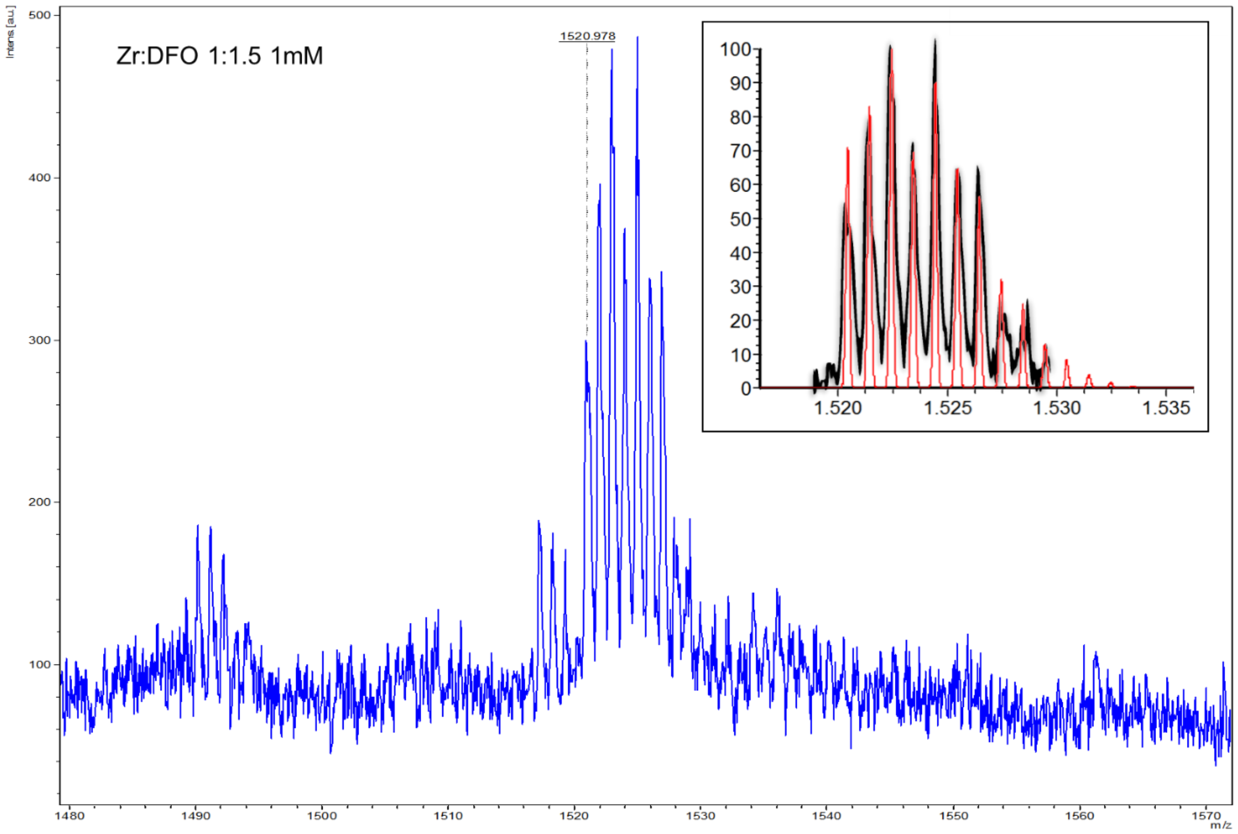

Figure S4. Detail of MALDI-TOF/TOF spectra of Zr:Desferrioxamine 1:1.5 highlighting the peak at m/z 1520.87. Inset: experimental (black) vs calculated (red) intensity distribution for $[KZr_2(DFO)_2HCCA(-2\ H^+)]^+$, $C_{60}H_{95}N_{13}O_{19}KZr_2$, calculated m/z 1520.46. HCCA (α-cyano-4-hydroxycinnamic acid) from the matrix.

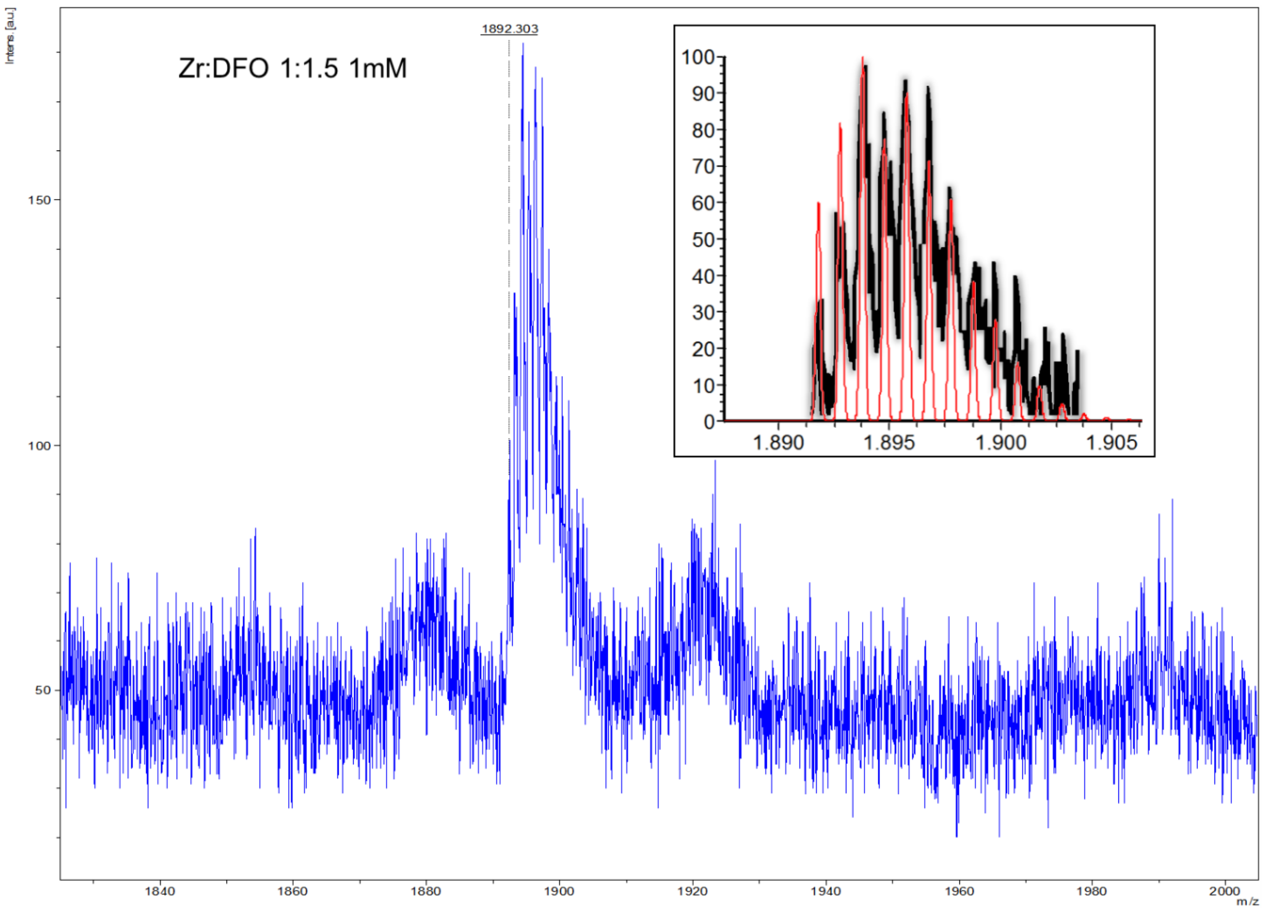

Figure S5. Detail of MALDI-TOF/TOF spectra of Zr:Desferrioxamine 1:1.5 highlighting the peak at m/z 1892.30. Inset: experimental (black) vs calculated (red) intensity distribution for $[HKZr_2(DFO)_3]^+$, $C_{75}H_{136}N_{18}O_{24}KZr_2$, calculated m/z 1891.77.

ESI analysis

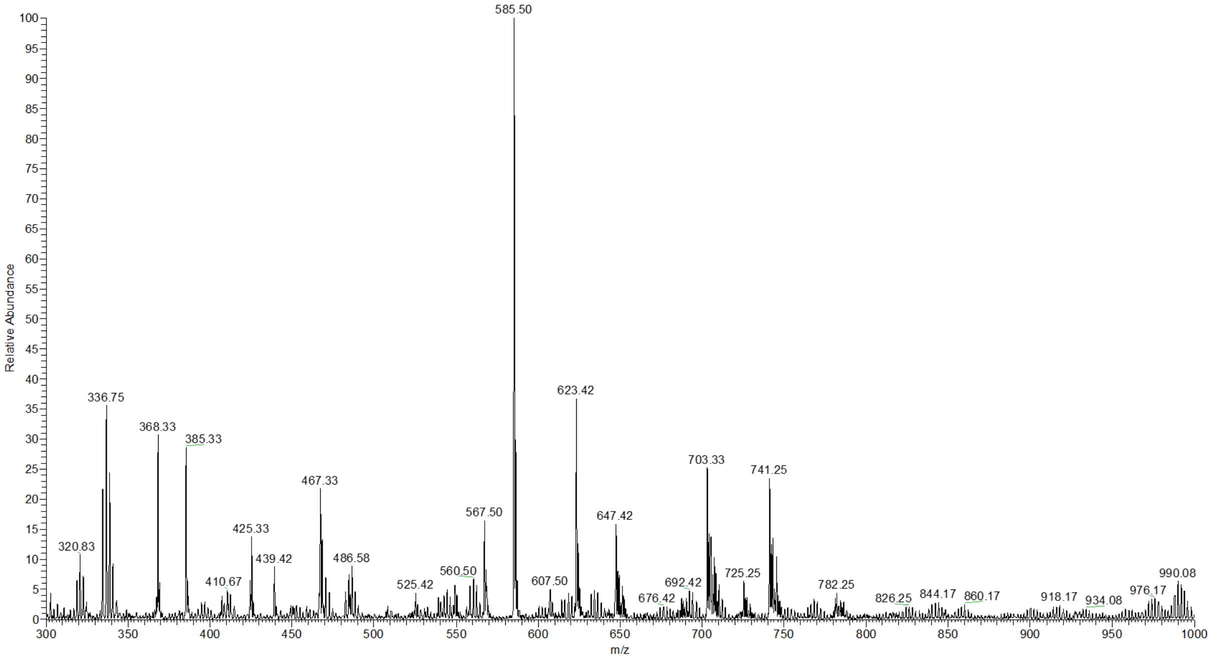

Figure S6. ESI mass spectrum of the Zr:DFO 1:1 1mM solution. Peaks at m/z 585.50 ($[NaH_3DFO]^+$), and 647.42 ($[ZrDFO]^+$, $C_{25}H_{45}N_6O_8Zr$, calculated m/z 647.23) are clearly visible.

Small-angle X-ray scattering (SAXS) experiments

SAXS experiments were performed to evaluate the size and the shape of the Zr-DFO complex in solution. In particular, aqueous solutions of Zr^{4+} and DFO in 1:1 molar ratio (0.08 M and 0.008 M) at pH 4 were prepared for the analysis.

Experimental setup

Small-angle X-ray scattering experiments were performed at 25 °C with a HECUS S3-MICRO SWAXS-camera (Kratky) equipped with a position-sensitive detector (OED 50 M, 1024 channels, 54 µm wide). Cu Kα radiation of wavelength λ = 1.542 Å was provided by a GeniX X-ray generator operating at a maximum power of 50 W. The sample-to-detector distance was 281 mm. The Kratky camera was calibrated in the small angle region using silver behenate (d = 58.38 Å).[9] The volume between the sample and the detector was kept under vacuum during the measurements to minimize the scattering from air. The sample was contained in a 2.0 mm thick borosilicate capillary tube sealed with hot melting glue. Scattering length density values[10] (SLD) were calculated on the basis of the chemical composition and the molecular volume of the complexes. Volumes were estimated using the 3V program[11] assuming Zr-DFO complexes with ratio 1:1, 2:2 and 2:3. The values of SLD and the molecular volume of these complexes are listed in Table S3.

Table S3. Molecular volume and SLD of the complexes formed by Zr^{4+} with desferrioxamine (H3DFO) with the ratios 1:1, 2:2 and 2:3.

**Zr-DFO complex**	**1:1**	**2:2**	**2:3**
V_{mol} [$Å^3$]	973	1951	2890
SLD [$Å^{-2}$]	$9.93*10^{-6}$	$9.93*10^{-6}$	$1*10^{-5}$

Simulated and experimental scattering profiles of Zr-DFO complexes

The theoretical SAXS curve of a macromolecule with a known atomic structure can be simulated using the CRYSOL program[12] which uses multipole expansion for fast calculation of the spherically averaged scattering pattern taking into account the hydration shell. This program can be used giving as an input the atomic coordinates (i.e. PDB file) of the molecule to predict the scattering curve or combined with the SAXS experimental data to minimize the discrepancy with

the theoretical curve using only two free parameters, the average displaced solvent volume per atomic group and the contrast of the hydration layer.

The parameters used for the simulation of the scattering profiles of Zr-DFO complexes with the ratios 1:1, 2:2 and 2:3 are listed below:

- maximum order of harmonics: 15
- order of Fibonacci grid: 17
- maximum scattering vector: 0.5 Å^{-1}
- number of points in the theoretical curve: 51
- solvent density (water): 0.334 e/Å^3
- contrast of hydration shell: 0.03 e/Å^3

Figure S7 shows the experimental scattering curve of the aqueous solution of Zr^{4+} and DFO in 1:1 molar ratio (0.08 M) at pH 4 along with the predicted scattering profiles of the 1:1, 2:2 and 2:3 complex forms obtained using CRYSOL.

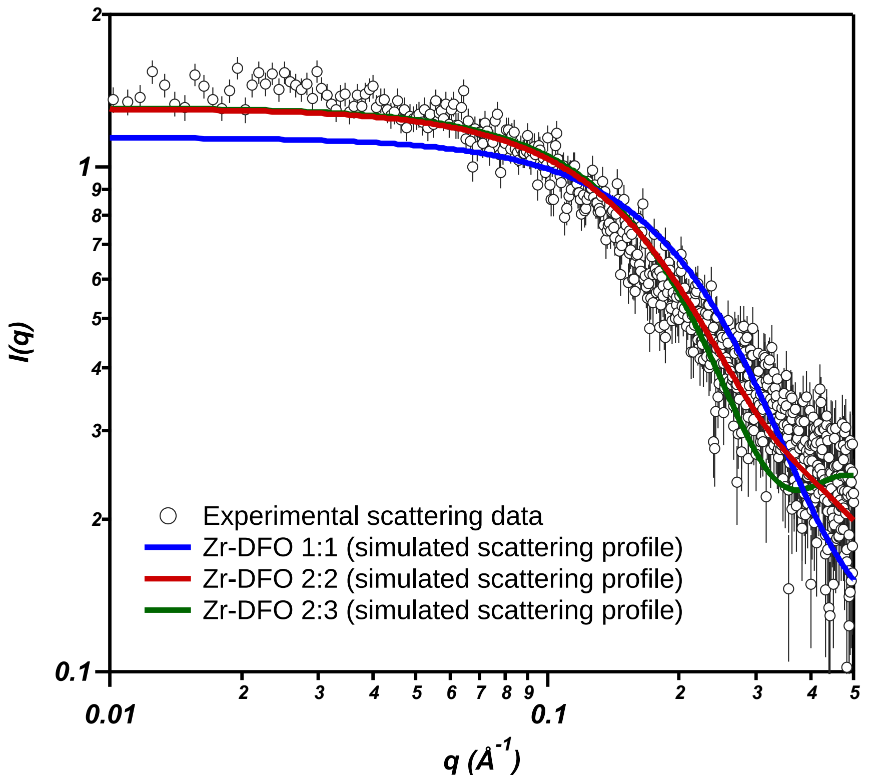

Figure S7. Experimental scattering curve of the aqueous solution of Zr^{4+} and DFO in 1:1 molar ratio (0.08 M) at pH 4 (empty circles), simulated scattering profiles of Zr-DFO complexes 1:1 (blue line, χ^2 = 4.05), 2:2 (red line, χ^2 = 1.92) and 2:3 (green line, χ^2 = 2.76) obtained by CRYSOL.

The results show that the simulated scattering profile of the Zr-DFO 1:1 complex is quite different from our experimental data, while a good agreement can be found if we consider the 2:2 and 2:3 simulated profiles. In particular, the best agreement in terms of χ^2 is found in the case of the 2:2 complex. This suggests that Zr-DFO complex, in the conditions probed by the SAXS experiment, is present mainly as a 2:2 specie.

Moreover, it can be seen that the experimental scattering pattern can be well modeled using a barbell form factor (see below).[13] Figure S8 shows the experimental scattering curve of the Zr^{4+} and DFO in 1:1 molar ratio (0.08 M) water solution along with the fitting obtained by using the barbell form factor and the simulated scattering profiles of the Zr-DFO 2:2 complex obtained by the CRYSOL program.

Figures S8 suggest that Zr-DFO complex in water in 1:1 molar ratio (0.08 M) at pH 4 can be modeled as a barbell form factor where the two poles of the barbell represent the two zirconium atoms of the 2:2 complex that are responsible for the main contribution to the measured scattering. The diameter and the length of the cylinder and the diameter of the two end caps obtained from the fitting are 4.4±0.3, 2.5±1.6 and 14.7±0.2 Å, respectively. Figure S9 reports the

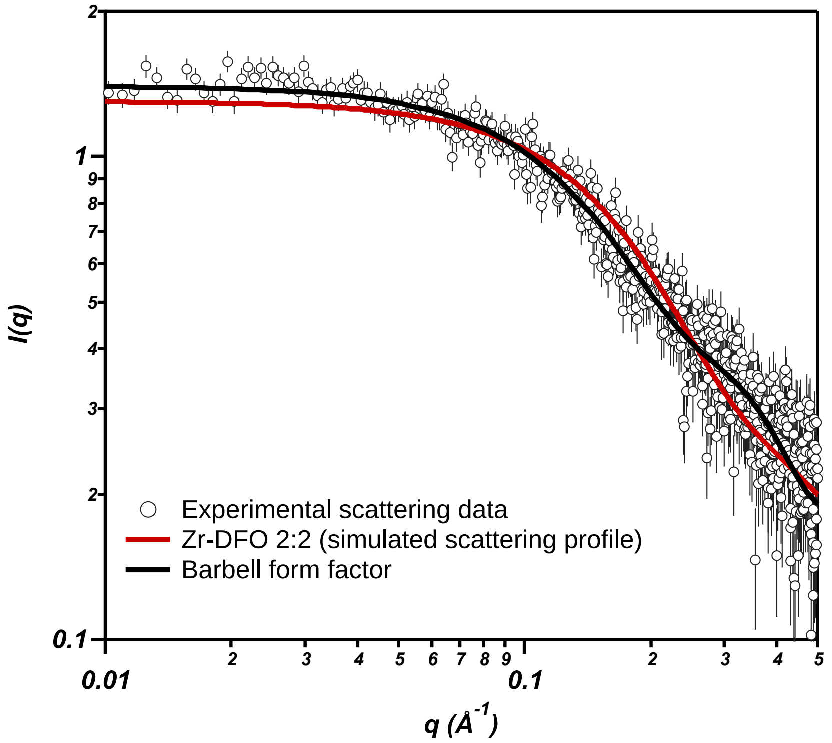

Figure S8. Experimental scattering curve of the aqueous solution of Zr^{4+} and DFO in 1:1 molar ratio (0.08 M) at pH 4 (empty circles), fitting with a barbell form factor (black line, χ^2 = 1.18) and simulated scattering profile of Zr-DFO 2:2 complex (red line, χ^2 = 1.92) obtained by CRYSOL.

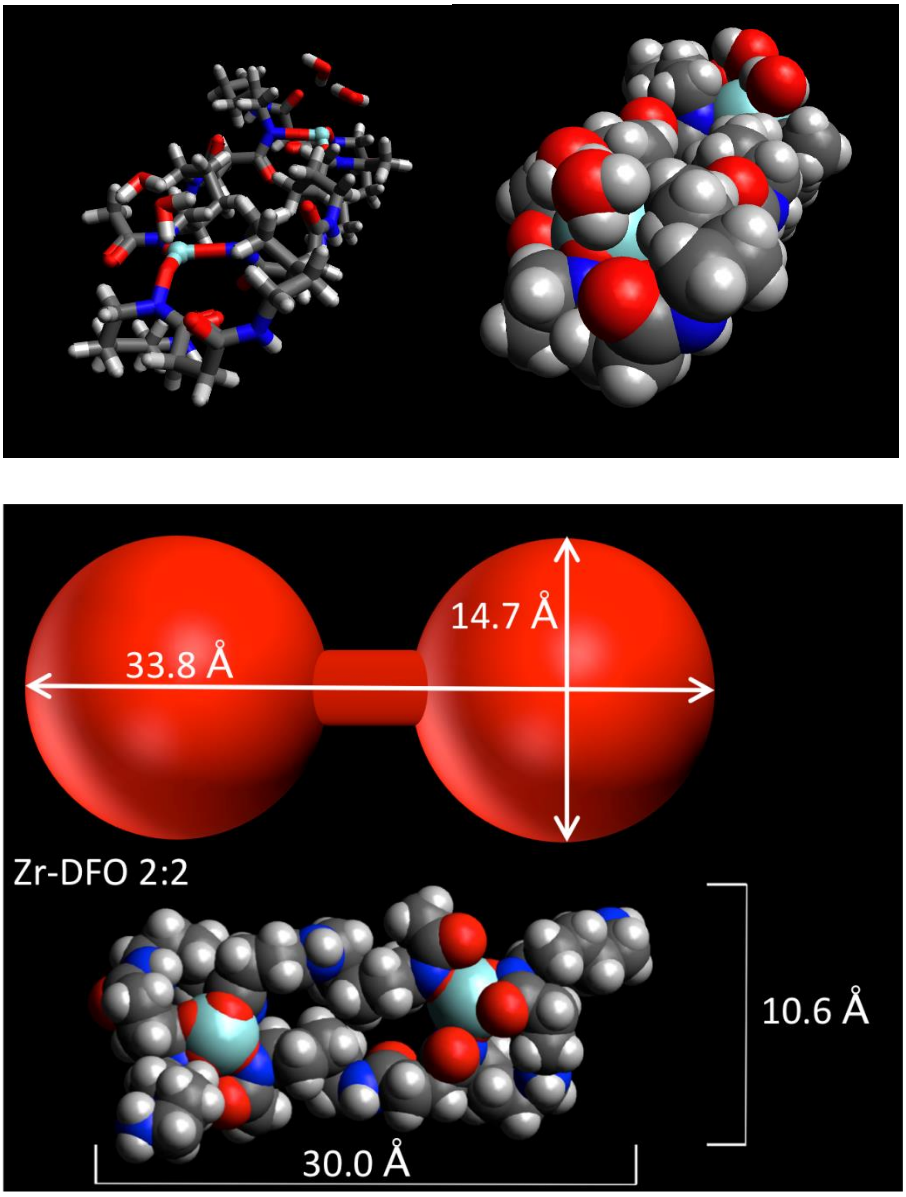

Figure S9. Ball and stick and CPK models of the simulated dimer case (top). Dimensions obtained with the barbell model and with the simulated Zr-DFO 2:2 structure (bottom).

comparison between the dimensions obtained with the barbell model and those of the modelled Zr-DFO 2:2 structure.

A model-independent estimation of the size (i.e., gyration radius, R_g) of the scattering objects in solution can be given by the Guinier approximation, according to equation S1:

$$ln(I(q)) = ln(I_0) - \left(\frac{R_g^3}{3}\right) q^2 \qquad \text{(S1)}$$

This approximation is valid when $q_{max} \leq 1/R_g$ (ref. 10).

Figure S10 shows the comparison of the Guinier plots (i.e. ln(I) vs q^2) obtained from the experimental SAXS data and simulated Zr-DFO 1:1, 2:2 and 2:3 scattering profiles.

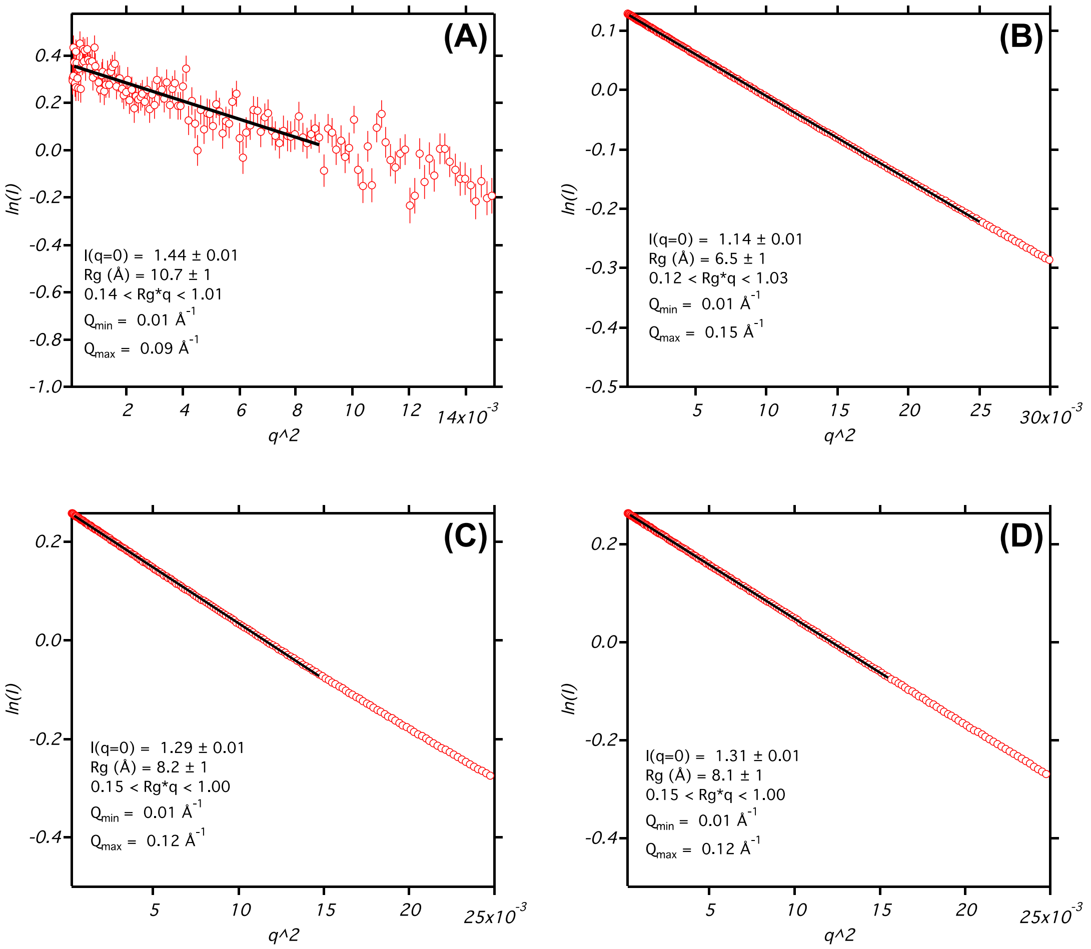

Figure S10. Guinier plots of experimental (A), simulated Zr-DFO 1:1 (B), 2:2 (C) and 2:3 (D) scattering profiles.

R_g for experimental data results 10.7±1.0 Å, which is not compatible with the mean size obtained from the Zr-DFO 1:1 case. The values obtained for all the simulated scattering curves are smaller than the experimental one: this can be probably ascribed to an underestimation of the hydration shell in the simulated models with respect to the real case. Considering the experimental uncertainty, R_g extracted from the SAXS experiment is closer to those obtained for the 2:2 and 2:3 simulated curves (≅ 8.2±1.0 Å) than to the 1:1 complex (R_g = 6.5±1.0 Å). This result, together with the good agreement of the experimental scattering curve with that obtained from the dimer simulation, further confirms the preferential formation of Zr-DFO 2:2 complex in aqueous solutions of Zr^{4+} and DFO in 1:1 molar ratio (0.08 M) at pH 4.

Barbell form factor

The barbell form factor is related to a sphereocylinder with spherical end caps with uniform scattering length density[13] (see Figure S11). The radius of the end caps is larger than that of the cylinder.

$$P(q) =< A^2(q) >$$

The brackets denote an average of the structure over all orientations.

Where

$$A(q) = \pi r^2 L \frac{\sin[(QL/2)\cos\vartheta]}{(QL/2)\cos\vartheta} \frac{2J_1(Qr\sin\vartheta)}{Qr\sin\vartheta} + 4\pi R^3 \int_{-h/R}^{1} dt \cos[Q\cos\vartheta(Rt + h + L/2)] \cdot (1 - t^2) \frac{J_1\left[QR\sin\vartheta\,(1-t^2)^{1/2}\right]}{QR\sin\vartheta\,(1-t^2)^{1/2}}$$

The volume of the barbell is:

$$V = \pi r_c^2 L + 2\left[\pi\left(\frac{2R^3}{3} + R^2 h - \frac{h^3}{3}\right)\right]$$

The fitting parameters are:

- scale
- cylinder radius, r_c (Å)
- cylinder length, L (Å)
- end cap radius, R (Å) where ($R \geq r_c$)
- SLD barbell (Å^{-2})
- SLD solvent (Å^{-2})
- bkg

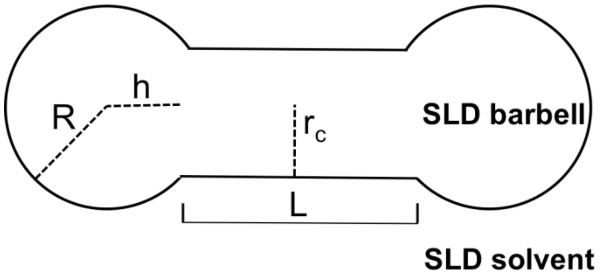

Figure S11. Schematic representation of a barbell with uniform scattering length density.

References

1) C. Bazzicalupi, A. Bianchi, T. Biver, C. Giorgi, S. Santarelli, M. Savastano, *Inorg. Chem.* **2014**, *53*, 12215–12224.

2) G. Gran, *Analyst* (London) **1952**, *77*, 661–671.

3) P. Gans, A. Sabatini, A. Vacca, *Talanta* **1996**, *43*, 1739−1753.

4) S. Dhungana, P. S. White, A. L. Crumbliss, *J. Biol. Inorg. Chem.* **2001**, *6*, 810-818.

5) Schrödinger Suite Release 2016-1, Schrödinger, LLC, New York, NY, **2016**: (a) Maestro v.10.5; (b) Jaguar, v. 9.1.Jaguar, v. 9.1.

6) F. Guerard, Y.-S. Lee, R. Tripier, L. P. Szajek, J. R. Deschamps, M. W. Brechbiel, *Chem. Commun.* **2013**, *49*, 1002-1004.

7) S. Dhungana, P. S. White, A. L. Crumbliss, *J. Am. Chem. Soc.* **2003**, *125*, 14760-14767.

8) J. P. Holland, V. Divilov, N. H. Bander, P. M. Smith-Jones, S. M. Larson, J. S. Lewis, *J. Nucl. Med.* **2010**, *51*, 1293-1300.

9) T. N. Blanton, T. C. Huang, H. Toraya, C. R. Hubbard, S. B. Robie, D. Louer, H. E. Göbel, G. Will, R. Gilles, T. Raftery, *Powder Diffr.* **1995**, *10*, 91–95.

10) L. A. Feigin, D. I. Svergun, *Structure Analysis by Small-Angle X-Ray and Neutron Scattering*, Plenum Press, New York, **1987**.

11) N. R. Voss, M. Gerstein, *Nucleic Acids Res.* **2010**, 1–8.

12) D. Svergun, C. Barberato, M. H. Koch, *J. Appl. Crystallogr.* **1995**, *28*, 768–773.

13) H. Kaya, *J. Appl. Crystallogr.* **2004**, *37*, 223–230.
